# Lateral Flow Assay to Detect Carbonic Anhydrase IX in Seromas of Breast Implant-Associated Anaplastic Large Cell Lymphoma

**DOI:** 10.3390/cancers17142405

**Published:** 2025-07-21

**Authors:** Peng Xu, Katerina Kourentzi, Richard Willson, Honghua Hu, Anand Deva, Christopher Campbell, Marshall Kadin

**Affiliations:** 1 Department of Pathology, University of Virginia, Charlottesville, VA 22903, USA; px3x@virginia.edu; 2 William A. Brookshire Department of Chemical and Biomolecular Engineering, University of Houston, Houston, TX 77204, USA; edkourentzi@uh.edu (K.K.);; 3 Department of Plastic and Reconstructive Surgery, Macquarie University, Sydney, NSW 2000, Australia; helen.hu@mq.edu.au (H.H.);; 4 Department of Plastic Surgery, Maxillofacial and Oral Health, University of Virginia, Charlottesville, VA 22903, USA; CAC5RB@uvahealth.org

**Keywords:** lateral flow assay, BIA-ALCL, Carbonic Anhydrase IX, seroma

## Abstract

Background/Objective: Breast implant-associated anaplastic large cell lymphoma (BIA-ALCL) has affected more than 1700 women with textured breast implants. About 80% of patients present with fluid (seroma) around their implant. BIA-ALCL can be cured by surgery alone when confined to the seroma and lining of the peri-implant capsule. To address the need for early detection, we developed a rapid point of care (POC) lateral flow assay (LFA) to identify lymphoma in seromas. Methods: We compared 28 malignant seromas to 23 benign seromas using both ELISA and LFA. LFA test lines (TL) and control lines (CL) were visualized and measured with imaging software and the TL/CL ratio for each sample was calculated. Results: By visual exam, the sensitivity for detection of CA9 was 93% and specificity 78%, while the positive predictive value was 84% and negative predictive value 90%. Quantitative image analysis increased the positive predictive value to 96% while the negative predictive value reduced to 79%. Conclusions: We conclude that CA9 is a sensitive biomarker for detection and screening of patients for BIA-ALCL in patients who present with seromas of unknown etiology. The CA9 LFA can potentially replace ELISA, flow cytometry and other tests requiring specialized equipment, highly trained personnel, larger amounts of fluid and delay in diagnosis of BIA-ALCL.

## Simple Summary

More than 1700 women worldwide with textured breast implants have developed a unique T-cell lymphoma (BIA-ALCL) in seroma fluid around their implant (s). Early detection is essential to plan definitive surgery before spread to adjacent tissues and lymph nodes. Carbonic Anhydrase IX (CA IX) was found to be significantly increased in fluids containing lymphoma compared to fluids of more common benign causes such as capsular contracture or implant leakage. We developed a rapid point of care paper-based lateral flow assay (LFA) to detect CA IX in seroma fluid that can be performed without specialized training similar to COVID-19 or pregnancy tests. Because CA IX is released by cells of multiple different epithelial cancers, this CA IX LFA has potential for broader application to cancer detection and treatment.

## 1. Introduction

Carbonic anhydrases (CAs) are ubiquitous metalloenzymes that catalyze the interconversion of carbon dioxide (CO_2_) and water (H_2_O) to bicarbonate (HCO_3_^−^) and protons (H^+^) [1]. Carbonic Anhydrase IX (CA9) is a hypoxia-inducible gene that has been associated with poor outcome in several cancers [2,3,4]. CA9 regulates intracellular and extracellular pH, influencing cancer cell survival and growth in acidic microenvironments [5,6]. Tumor-associated CA9 has two major forms. One is a cell-associated, transmembrane protein seen on Western blots as a twin band of 54/58 kDa, expressed in gastric mucosa and in several types of cancer. The other is a soluble protein s-CA9 of 50/54 kDa, which is released into the culture medium or into the body fluids, most likely by proteolytic cleavage of the extracellular part from transmembrane and intracellular sequences [7]. CA9 expression in non-cancerous tissues is generally confined to epithelia of the stomach, gallbladder, pancreas and intestine [8].

BIA-ALCL is an ALK-, CD30+ T-cell lymphoma occurring rarely in patients with textured breast implants. Patients with BIA-ALCL commonly present with breast swelling due to accumulation of fluid (seroma) around one or both implants [9]. A recent study using ELISA showed significantly higher levels of CA9 in 10 malignant seromas compared to 3 benign seromas [10]. While suitable for proof of concept, ELISA requires a plate reader and use of standard curves, batching of specimens for economy and hours to complete. Our objective is to develop a point of care lateral flow assay (LFA) that can be performed without specialized equipment in less than 20 min. We compared 28 malignant seromas to 23 benign seromas using both ELISA and an in-house-developed LFA for CA9. A heatmap showing CA9 clustered with other biomarkers of BIA-ALCL (CD30, IL-9, IL-10, IL-13) distinguished nearly all malignant from benign seromas.

## 2. Materials and Methods

### 2.1. Seroma Samples and Cell Lines

Seroma fluids were obtained from 51 women in the USA and Australia. including 28 with a pathological diagnosis of BIA-ALCL and 23 with benign seromas. The study was approved by the Institutional Review Boards of Rhode Island Hospital (#1325270-6) and the University of Virginia (2024-493). Upon receipt, seroma samples were used to prepare cytospins for morphology and immunohistochemistry, while the remaining material was stored at −80 °C or in liquid nitrogen for future research.

BIA-ALCL cell lines TLBR1 and TLBR2 generously provided by Dr. Alan Epstein (Department of Pathology, Keck School of Medicine, University of Southern California, Los Angeles, CA, USA) were cultured in RPMI medium supplemented with 10% fetal bovine serum and 100 IU/mL IL-2. After 48 h of incubation, cells were harvested by centrifugation, and the resulting supernatants were collected for lateral flow assay (LFA).

### 2.2. Preparation of LFA Strips and Conjugation of Latex Detection Particles with CA9 Antibodies

LFA strips were prepared by assembling CN95 membrane (Sartorius; cat# 1UN95ER100025NTB, Göttingen, Germany) on a 30 cm backing adhesive card (DCN Diagnostics; cat# MIBA-020, Carlsbad, CA, USA) along with an absorbent pad (Ahlstrom; cat# 440, Helsinki, Finland) and a sample pad (Ahlstrom; cat# 8980). The nitrocellulose membrane was striped with goat anti-CA9 antibodies (1 mg/mL; Bio-techne; Cat# AF2188, Minneapolis, MN, USA) and sheep anti-mouse control antibodies (1 mg/mL; Jackson ImmunoResearch Laboratories Inc. Cat# 515-005-003, West Grove, PA, USA) using a BioDot dispenser (BioDot; Cat# XYZ30600124, Irvine, CA, USA) as reported previously [11]. Detection mouse anti-CA9 antibody (Bio-techne; cat# MAB2188) was conjugated onto carboxylated red latex particles (Thermo Fisher, Cat# DR1040CA, Waltham, MA, USA) as reported [11].

### 2.3. Lateral Flow Assay (LFA)

The dynamic range of detection was determined with recombinant human CA9 (Bio-techne Cat #: 2188-CA-010; 0–50 ng/mL) spiked into a benign seroma fluid. Supernatants of 48 h cell cultures of BIA-ALCL lines TLBR1 and TLBR2 were analyzed as positive controls.

LFA assay of clinical seromas was performed following the previously published protocol [11]. Briefly, seroma samples were diluted at a 1:3 ratio in running buffer (1× Phosphate-buffered saline with 0.2% Triton and 0.5% BSA). Antibody-conjugated detection particles were diluted by a ratio of 1:20 in running buffer and sonicated for 30 s. A total of 30 μL of diluted seroma was loaded onto the sample pad. After 5 min, 20 μL of diluted detection antibody was added to each strip. Each strip was then washed with 20–60 μL of running buffer after an additional 5 min.

### 2.4. Image Analysis

LFA strips were photographed with an imager (Bio-Rad ChemiDoc Touch Image System, Hercules, CA, USA), and image analysis was performed using NIH Image J software (Fiji). The density of the test line (TL) and the control line (CL) was measured, and TL/CL ratios were calculated and compared.

### 2.5. Immunostaining

Cells were cytospun at 1000 rpm for 5 min and subsequently fixed in 4% paraformaldehyde (PFA) for 5 min. Fixation was stopped with a 10 min wash in 100 mM Tris·HCl (pH 8.0). Cells were then blocked for 30 min using Odyssey blocking buffer (Licor, Cat# 927-40003). Goat anti-CA9 antibody (Bio-techne, Cat#: AF2188) was diluted 1:200 in blocking buffer and applied to the cells for overnight incubation. Following three washes with PBS, cells were incubated with donkey anti-goat Alexa Fluor 488 secondary antibody (Thermo Fisher, Cat#: A11055). Nuclear staining was performed using Hoechst 33342 (Thermo Fisher, Cat#: 735969; 1:2000 dilution). After 3 times of PBS wash, cells were mounted, and images were captured using the Cytation 5 imaging system (BioTek Instruments, Winooski, VT, USA).

### 2.6. Enzyme Linked Immunosorbant Assay (ELISA)

BIA-ALCL samples were diluted in 1:100 and 1:200. Benign samples were loaded directly. Each sample was tested in duplicate. ELISA for CA9 was performed with a kit from Bio-techne (Cat#, CAD900) Newark, Delaware. ELISA for CD30 was performed with a kit from Bio-techne (Cat#, DY6126-05) using standard curves according to instructions from the manufacturers.

### 2.7. Statistical Analysis

Data are expressed as mean ± SD. Comparisons among more than two groups were assessed using repeated measures or factorial ANOVA, followed by Sidak’s post hoc test, performed with GraphPad Prism version 10.4 (GraphPad Software, La Jolla, CA, USA). Student’s *t*-test was used for comparisons between two groups, while categorical data were analyzed using the chi-square test.

Sensitivity refers to the ability of the test to detect positive cases, and specificity reflects the test’s ability to exclude negative cases. Sensitivity = true positives/(true positives + false negatives) and specificity = true negatives/(true negatives + false positives) were calculated to assess assay performance. False positives are benign cases with a positive test line; false negatives are BIA-ALCL with no test line. Positive Predictive Value (PPV) and Negative Predictive Value (NPV) were also determined to evaluate the diagnostic accuracy. PPV was calculated as true positives/(true positives + false positives). NPV was calculated as true negatives/(true negatives + false negatives).

Receiver operating characteristic (ROC) curves were constructed to evaluate the diagnostic performance of CA9 by analyzing sensitivity and specificity across a range of threshold values. The area under the curve (AUC) was calculated using GraphPad Prism software, serving as a quantitative indicator of the assay’s overall diagnostic accuracy. Higher AUC values reflect improved performance, with values approaching 1 indicating excellent distinction between malignant and benign samples.

## 3. Results

### 3.1. CA9, CD30, and Cytokines Differentiate BIA-ALCL from Benign Seromas

**Figure 1:**
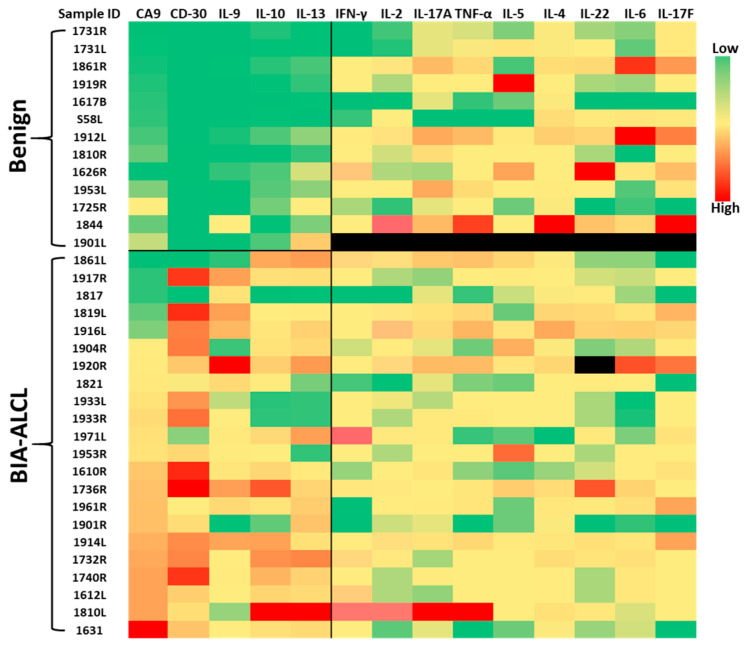
Heatmap comparing concentrations of CA9, CD30 and 12 cytokines in BIA-ALCL and benign seromas.

We compared the concentrations of CA9, CD30, and a panel of 12 cytokines in 22 BIA-ALCL seroma samples and 13 benign samples, including 5 bilateral seromas from five patients using ELISAs for CA9 and CD30 and a Biolegend Human 12-plex T helper (Th) Cytokine Panel as previously reported [11]. Representative results are visualized in a heat map (Figure 1). Most patients with benign seromas had capsular contracture, a non-malignant postoperative complication. In one case (1933), both left and right sides had high levels of CA9 and CD30 with positive LFAs indicating bilateral BIA-ALCL, although pathologists originally interpreted the left side as benign, suggesting that LFA results can guide re-evaluation of pathology. Overall, benign seromas displayed consistently low expression levels—indicated by green coloring—of CA9, CD30, IL-9, IL-10 and IL-13, whereas these markers were markedly elevated in the majority of BIA-ALCL samples. This clear differential expression highlights their potential clinical utility as biomarkers for distinguishing malignant from benign seromas.

Clinically, 26 of the 29 BIA-ALCL patients were diagnosed at an early stage (stage IA), with only 3 stage IIA (s62, 1610, 1722) and none with higher stage disease. As shown in Table 1, there was no significant difference in mean age or implant duration between patients with BIA-ALCL and those with benign seromas. Interestingly, a significant difference was observed for BIA-ALCL and higher-grade (Grade 3 or 4) textured implants, particularly those manufactured by Silimed/Amostra and Allergan. In contrast, current data on breast implant-associated squamous cell carcinoma (BIA-SSC) cases do not show a significant difference between smooth and textured implants [12].

Collectively, these data demonstrate feasibility of detection of early-stage BIA-ALCL and the potential value of biomarker-based assays in the timely and accurate diagnosis of BIA-ALCL. These tools could play a pivotal role in clinical decision-making and patient management, especially in guiding further diagnostic workup in individuals with indeterminate or suspicious seroma presentations.

### 3.2. Analytical Sensitivity of CA9 LFA

**Figure 2:**
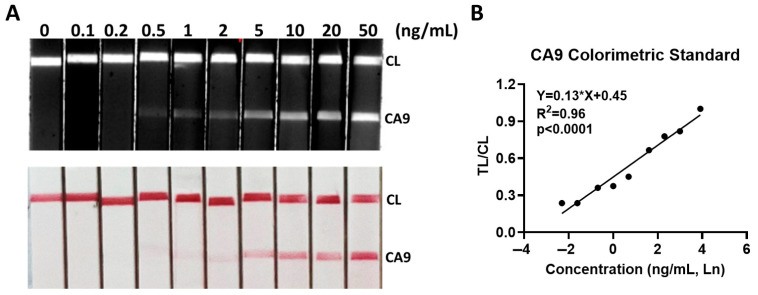
Dynamic range of CA9 detection using LFA strips. (**A**) Nine test solutions were prepared by spiking recombinant CA9 into benign seroma samples at defined concentrations, using a 1:2 ratio of seroma to running buffer. Results were imaged using both an iPhone camera (color) and a Bio-Rad imager (black and white) and image analysis was performed using NIH Image J software. (**B**) The TL/CL ratio exhibited a strong linear correlation with CA9 concentration (Y = 0.13X + 0.45, r^2^ = 0.96, *p* < 0.0001). CL, control line; TL (CA9), test line.

The lower limit of detection (LOD) for the CA9 LFA was established at 500 pg/mL using recombinant CA9 protein spiked into a benign seroma sample, as shown in Figure 2A. This concentration represents the lowest amount of CA9 that the assay can consistently and reliably detect under controlled experimental conditions. Images of the test strips were captured using both a Bio-Rad imaging system (black and white) and an Iphone camera (color). The Bio-Rad imager demonstrated higher sensitivity compared to the standard phone camera or visual inspection. Quantitative analysis revealed a strong correlation between the test line/control line (TL/CL) intensity ratio and CA9 concentration, with an R^2^ value of 0.96 using linear regression (X-axis representing CA9 concentration) (Figure 2B). This supports the LFA’s potential for semi-quantitative analysis when coupled with digital imaging.

### 3.3. CA9 Expression in TLBR BIA-ALCL Cell Lines

**Figure 3:**
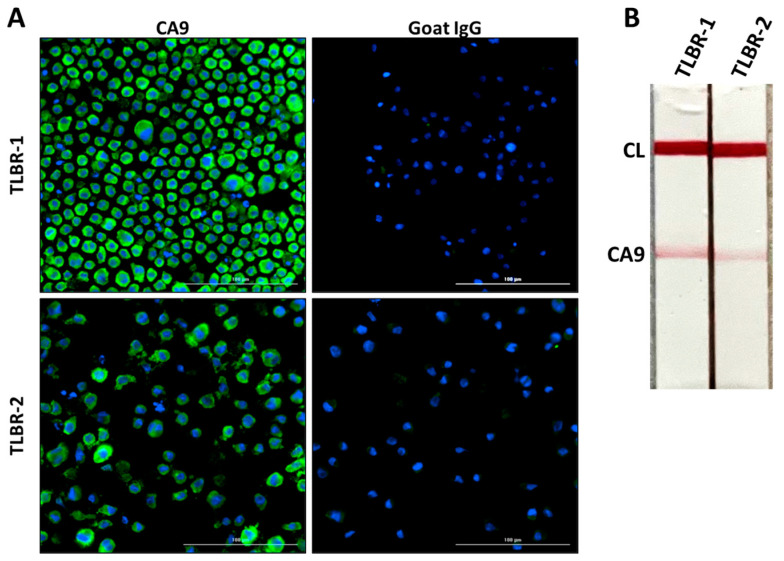
CA9 Expression in TLBR-1 and TLBR-2 Cell Lines. (**A**) Immunofluorescence staining of TLBR-1 and TLBR-2 cell lines showed CA9 expression (green), with nuclei counterstained with DAPI in blue. Goat IgG was used as a negative control. (**B**) LFA analysis demonstrated the presence of CA9 in culture supernatants of both cell lines. CL, control, line; Scale bar = 100 μm.

TLBR-1 and TLBR-2 are two cell lines derived from BIA-ALCL that are known to express CA9 [10]. As an initial validation step, we confirmed that CA9 was present on the membranes of both TLBR-1 and TLBR-2 cell lines, with TLBR-1 exhibiting a greater fluorescence than TLBR-2 (Figure 3A). The CA9 LFA was then tested for ability to detect CA9 in supernatants from these cell lines. The presence of CA9 in supernatants was confirmed by clear test line signals on the LFA strip, as shown with an iPhone camera (color) in Figure 3B. These findings demonstrate that the LFA reliably detects CA9 in biologically relevant samples and supports its potential use in clinical screening applications.

### 3.4. Detection of CA9 in Clinical Seroma Samples

**Figure 4:**
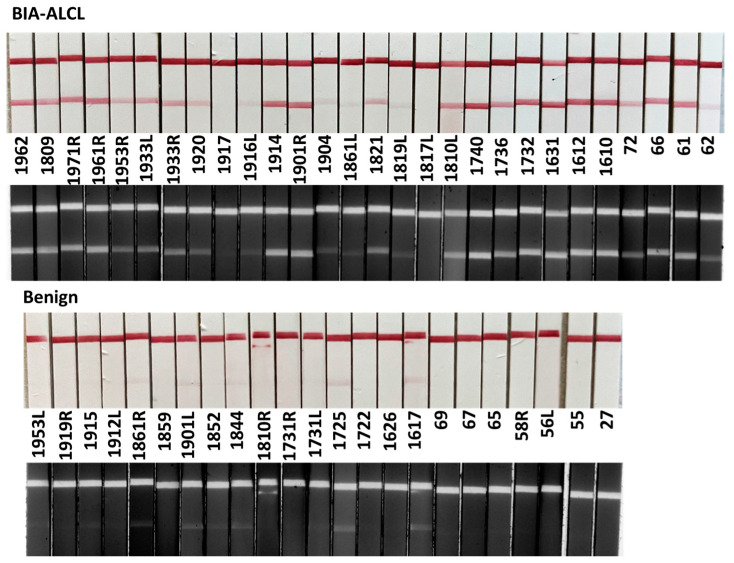
CA9 LFA analysis on benign and BIA-ALCL seromas. Images were captured using an iPhone camera (color) and the Bio-Rad imager with the Alexa Fluor 488 channel (black and white). Density of CL and TL were determined from Bio-Rad images.

In clinical testing with visual inspection of test strips (Figure 4), the CA9 LFA detected BIA-ALCL in 26 of 28 malignant seromas. One malignant sample (1861L) produced a weakly positive test band even at a 1:3 dilution, with a corresponding ELISA-measured CA9 concentration around 400 pg/mL. This suggests that the LFA could detect CA9 at levels below 200 pg/mL in this case, indicating high analytical sensitivity. However, two malignant samples (1817L and 1917) were not detected by the CA9 LFA, representing false-negative results. These findings highlight the need to investigate additional factors that may affect CA9 expression or assay performance in certain malignant samples.

Conversely, four benign samples (1617, 1725, 1844, 1901L) yielded faint CA9 test lines, representing false-positive results. Patients 1617 and 1725 had grade 3 surface-textured breast implants. Patient 1617 had CA9 of 607 pg/mL and patient 1725 had elevated CA9 at 4587 pg/mL. Patient 1844 had a microtextured grade 2 surfaced textured implant, moderate CA9 elevation (1297 pg/mL) and elevated IL-9. Patient 1901 had a clear positive CA9 TL with 82,600 pg/mL CA9 and 41,300 pg/mL CD30 on the right side, but on the left side a faint CA9 TL, 3099 pg/mL CA9 and CD30 609 pg/mL. Both sides had grade 3 surface textured implants. IL-13 was similarly elevated on both sides—9899 pg/mL on the right side and 8088 pg/mL on the left—raising the possibility of an evolving bilateral ALCL on the left side. These results suggest that further testing with ELISA for CA9 and CD30 would be useful in cases with faint CA9 test lines.

### 3.5. Image-Based Quantitative Analysis

**Figure 5:**
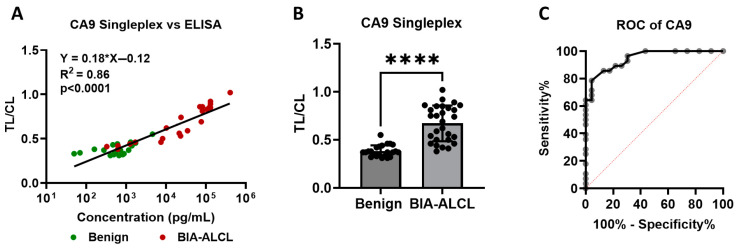
Statistical analysis of CA9 LFA performance. (**A**) A strong linear correlation was observed between CA9 concentrations measured by ELISA and the TL/CL ratio from the LFA (Y = 0.18X − 0.12, r^2^ = 0.86, *p* < 0.0001). (**B**) CA9 levels were significantly elevated in BIA-ALCL samples compared to benign seromas (Benign: 0.38 ± 0.06, n = 23; BIA-ALCL: 0.67 ± 0.19, n = 28; *t*-test, **** *p* < 0.0001). (**C**) Receiver Operating Characteristic (ROC) curve analysis yielded an area under the curve (AUC) of 0.95, indicating excellent diagnostic performance.

To assess the performance of the CA9 lateral flow assay (LFA) in a semi-quantitative manner, we conducted image-based analysis of test strips and digital imaging to calculate the intensity ratio of the test line to control line (TL/CL). These values were then compared to corresponding CA9 concentrations measured by ELISA in the same samples. Linear regression analysis revealed a strong and statistically significant correlation between TL/CL ratios and ELISA-measured CA9 concentrations (y = 0.18x − 0.12, R^2^ = 0.86, *p* < 0.0001; Figure 5A), indicating that the LFA can provide an accurate estimate of CA9 levels.

Importantly, TL/CL ratios in seroma samples from BIA-ALCL patients were significantly elevated compared to those from benign cases (Benign, 0.38 ± 0.06, n = 23; BIA-ALCL, 0.67 ± 0.19, n = 28; *t*-test, *p* < 0.0001; Figure 5B), highlighting a clear distinction in signal intensity between malignant and non-malignant samples. These results support the utility of the CA9 LFA not only as a qualitative screening tool but also as a potential semi-quantitative assay when paired with image analysis. This approach could enhance diagnostic confidence and allow for more nuanced clinical interpretation, especially in resource-limited settings where ELISA may not be feasible.

### 3.6. Diagnostic Performance of CA9 Lateral Flow Assay

The diagnostic performance of the CA9 lateral flow assay (LFA) was assessed using two evaluation methods: visual inspection and quantitative image analysis.

Based on visual inspection of the LFA test strips, the assay exhibited a sensitivity of 93% and a specificity of 78% in detecting BIA-ALCL cases. The positive predictive value (PPV) was 84% and NPV 90%. These results, summarized in Table 2, demonstrate that the visually interpreted CA9 LFA could serve as a useful non-invasive screening tool, particularly effective for excluding BIA-ALCL in low-risk patients.

Using quantitative image analysis, with a TL/CL intensity ratio cutoff of 0.5, the assay achieved a sensitivity of 79% and a specificity of 96%. This approach significantly improved the PPV to 84%, while reducing NPV to 79%, as shown in Table 3. These findings indicate that incorporating objective image-based metrics enhances diagnostic specificity and overall accuracy, helping to minimize false positives while preserving the assay’s ability to rule out disease.

Additionally, receiver operating characteristic (ROC) curve analysis for the CA9 LFA TL/CL determined by image analysis yielded an area under the curve (AUC) of 0.946 (Figure 5C), reflecting excellent diagnostic accuracy. Notably, this AUC value is nearly identical to those observed for IL-10 and IL-9 LFAs [11], suggesting that CA9 performs comparably to other promising biomarkers in distinguishing malignant from benign seromas.

Together, these results highlight the CA9 LFA as a robust and versatile diagnostic tool for BIA-ALCL, with potential applications for both point-of-care screening and more comprehensive clinical workflows.

## 4. Discussion

There are more than 35 million women worldwide living with breast implants with an estimated 1 in 3000–7000 risk of developing BIA-ALCL, an overall incidence of 2.81 per 1000 textured implant patients [13]. About 80% of patients present with breast swelling, pain and/or erythema associated with a unilateral effusion (seroma) confined to the capsule of a textured implant. Survival is significantly improved by detection of BIA-ALCL when remaining localized to seromas and/or the lining of the peri-implant capsule [14,15].

To address the need for early detection, we developed a rapid point of care LFA for CA9 released by BIA-ALCL tumor cells. Our study detected CA9 in 25 of 28 seromas of patients with in situ (stage IA) BIA-ALCL compared with 4 of 11 in situ samples and 7 tumor stage in the study by Oishi et al. [10]. There was a significant increase in implant surface texture grade of patients with BIA-ALCL compared with those having benign seromas. The majority of our patients with benign seromas had capsular contracture, a frequent complication of breast implants. By visual inspection, potentially useful in the clinic, sensitivity for detection of BIA-ALCL with the CA9 LFA was 93%, specificity 78%, PPV 84% and NPV 90%. These results suggest the CA9 LFA is a good screening test to detect BIA-ALCL in patients with early disease confined to seroma and surrounding capsule. False positives would be mitigated by further testing.

Longo et al. recommended that in the presence of peri-implant fluid accumulation, ultrasound-guided fine-needle aspiration should be performed in an appropriate healthcare facility. Fresh aspiration fluid should be submitted as soon as possible, preferably within 12 h, to the pathology reference center and a part of the collected fluid must be sent to the microbiology laboratory for cultural examination [16]. Current best practice guidelines for diagnosis of BIA-ALCL include demonstration of atypical/anaplastic cells that express CD30 by immunohistochemistry or flow cytometry [17]. These tests require 20–50 mL of fluid, specialized equipment and pathologists familiar with BIA-ALCL for interpretation, resulting in delay of diagnosis, multiple patient visits and increased costs. Our LFA requires less than 1 mL of fluid and no more than 20 min for visual interpretation. LFA during initial and follow-up visits or intra-operative evaluation of seromas will provide physicians with immediate feedback for patient management. Patients will be spared time away from work and family, anxiety with diagnostic uncertainty and morbidity due to delay of diagnosis with a higher likelihood of surgeons being able to make definitive pre-operative plans. LFAs can potentially be performed at local physician practices, in rural and underserved areas at less cost, reducing the burden on national healthcare.

A limitation of the study was the small number of samples available from patients with this rare disease. Moreover, samples were obtained from multiple sources with likely differences in specimen handling. A positive LFA does not distinguish between in situ and infiltrative BIA-ALCL described by Laurent et al. [18]. All but three of our cases were in situ whereas Oishi et al. detected CA9 in mostly infiltrative cases [10]. Pathology was not available for two samples that were interpreted as benign but had a positive CA9 LFA. Nevertheless, the CA9 LFA had 93% sensitivity and 84% PPV by visual interpretation, and significant differences were shown between patients with BIA-ALCL and those with benign seromas. Thus, we conclude that CA9 LFA has potential for screening of patients with seromas of unknown etiology.

We speculate that this CA9 LFA has potential for screening of more common cancers [19]. For example, a major clinical problem is distinction of renal cancer from benign kidney nodules detected radiographically. CA9 is expressed on the surface of tumor cells in renal clear cell carcinoma, the most common form of renal cancer [20]. CA9 is an independent predictor of survival in advanced renal clear cell carcinoma [21]. Published ELISA studies detected CA9 in serum of patients with renal clear cell carcinoma. A CA9 LFA could potentially permit rapid detection of carcinoma in patients with nodules/masses of unknown etiology avoiding invasive procedures, e.g., kidney biopsies.

## 5. Conclusions

We conclude that the CA9 LFA is a practical and convenient option for early detection of BIA-ALCL. The sensitivity of 93% and NPV of 90% of the LFA by visual inspection has potential for rapid screening of patients with seromas of unknown etiology. This means that the CA9 LFA could be used in local physician offices and in rural or other remote underserved areas including non-industrialized countries where more extensive testing by referral laboratories and by specialized personnel including pathologists are not readily available. The potential low cost of the CA9 LFA could reduce patient care costs and the national health burden of participating countries.

## Figures and Tables

**Table 1:** Summary of Clinical Samples.

		Benign	BIA-ALCL	*p* Value
**Number of Subjects**		23	28	
**Age**		46.9 ± 10	48.7 ± 9	Ns
**Implant Duration**		7.8 ± 4.3	8.6 ± 4.3	Ns
**Implant Surface grade**	**4**	2	8	
	**3**	8	14	
	**2**	9	5	0.02 *
	**Unknown**	4	1	
**Implant Manufacturer**	**Silimed/Amostra**	2	8	
	**McGhan**	4	6	
	**Mentor**	5	4	
	**Natrelle/Allergan**	4	7	
	**Others**	6	2	0.01 *
	**Unknown**	2	1	

* Chi-Square test without unknown *p* < 0.05.

**Table 2:** Visual inspection.

CA9	BIA-ALCL	Benign	Total
**Positive**	26	5	31
**Negative**	2	18	20
**Total**	28	23	51
**Sensitivity**	93%	**PPV**	84%
**Specificity**	78%	**NPV**	90%

**Table 3:** Image analysis.

CA9	BIA-ALCL	Benign	Total
**Positive**	22	1	23
**Negative**	6	22	28
**Total**	28	23	51
**Sensitivity**	79%	**PPV**	96%
**Specificity**	96%	**NPV**	79%

## Data Availability

The data presented in this study are available on request from the corresponding author due to protecting patients’ personal information.

## Abbreviations

The following abbreviations are used in this manuscript:

LFA	Lateral Flow Assay
CA9	Carbonic Anhydrase IX
BIA-ALCL	Breast Implant Associated Anaplastic Large Cell Lymphoma
ELISA	Enzyme-Linked Immunosorbent Assay
PPV	Positive Predictive Value
NPV	Negative Predictive Value
